# Blocked Atrial Bi/Trigeminy In Utero Evolving in Supraventricular Tachycardia after Birth

**DOI:** 10.1155/2012/406497

**Published:** 2012-07-16

**Authors:** V. Martucci, A. Cerekja, A. Caiaro, G. Bosco, R. Lucchini, G. Piacentini, B. Marino, Flavia Ventriglia

**Affiliations:** ^1^Pediatric Cardiology, Sapienza University of Rome, Viale Regina Elena, 324, 00161 Rome, Italy; ^2^Ultrasound Division, ASL Roma B, 00169 Rome, Italy; ^3^Neonatal Pathology and NICU, Policlinico Umberto I, Sapienza University of Rome, 00161 Rome, Italy

## Abstract

Transient episodes of fetal bradycardia (heart rate less than 110 bpm) are usually benign and typically result from increased vagal stimulation in the fetus. Causes of sustained fetal bradycardia include sinus bradycardia, blocked atrial bigeminy/trigeminy, high-degree atrioventricular block, and long QT syndrome. We present the case of a 34-year-old Caucasian patient referred to our department for “blocked atrial bigeminy with pseudobradycardia” detected elsewhere at 33 weeks of gestation. A fetal echocardiography showed during all the examination a blocked atrial trigeminy with a mean fetal heart rate of 100 bpm. After birth three subsequent ECGs until day 3 showed no evidence of atrial extrasystoles, confirming the well-known frequent regression of this kind of fetal benign arrhythmia, but on day 11 recurrence of supraventricular trigeminy and development of episodes of paroxystic supraventricular tachycardia were observed. On the basis of this observation, we recommend that fetuses with complex atrial ectopic beats should be closely monitored before and after birth for evidence of new arrhythmias.

## 1. Introduction 

Fetal sinus bradycardia was defined before as a heart rate <100 bpm, but in 2009 [1] this threshold was revised to <110 bpm by the American College of Obstetrics and Gynecology in response to population data. Transient episodes of fetal heart rate of less than 110 bpm are usually benign and typically result from increased vagal stimulation in the fetus commonly associated with abdominal pressure by the ultrasound probe. Causes of sustained fetal bradycardia include sinus bradycardia, blocked atrial bigeminy/trigeminy, high-degree atrioventricular block, and long QT syndrome. 

Persistent atrial bigeminy and trigeminy with blocked premature beats may lower the average heart rate of the fetus to 70–100 bpm. This benign form of fetal bradycardia is the result of blocked premature atrial contractions occurring after one or two sinusal beats (resp., an atrial bigeminy or trigeminy) [2] that are not conducted to the ventricle and consequently nor to the aorta, probably due to the refractoriness of the A-V tissue. Subsequently a sinus pause is observed and a consequently fetal heart rate is low. The diagnosis can be reached by identifying the relationship between atrial and ventricular contraction using simultaneous recording in M-mode fetal echocardiography. 

Generally, this type of bradycardia is intermittent, does not require treatment, resolves spontaneously with advancing gestation or after birth, and is not usually associated with cardiac failure [3]. It occasionally may present as sustained bradycardia, which makes it difficult to differentiate from A-V block. However, blocked atrial bigeminy can be distinguished from sinus bradycardia or atrioventricular block by examination of the Doppler flow pattern in the inferior vena cava or hepatic veins, which shows flow reversal [4, 5]. 

Nevertheless, data regarding the prevalence, mechanisms, and the long-term outcome of fetuses with bradycardia are still limited. 

## 2. Case Report 

We present herein the case of a 34-year-old Caucasian patient G1P0 that was referred to our department for “blocked atrial bigeminy with pseudobradycardia” detected elsewhere at 33 of weeks of gestation. Furthermore, a placenta previa was diagnosed. 

**Figure 1:**
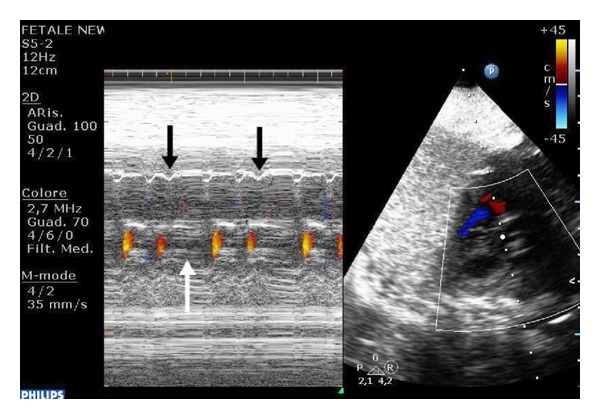
Color M-mode. Notice how every 2 atrial contractions lead to regular opening of the aortic valve, there is a premature atrial beat (black arrow) that is not conducted to the ventricles and does not lead to aortic valve opening (white arrow).

**Figure 2:**
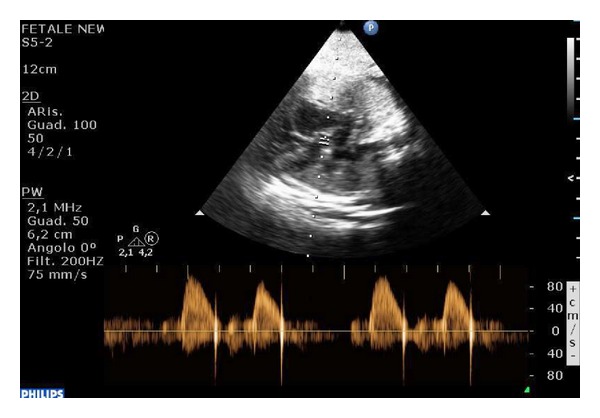
Pulsed Doppler wave of left ventricular outflow tract. There is a pause after 2 regular beats.

The patient was scanned at our department at 37 weeks of gestation. An echocardiography showed during all the examination an arrhythmia that in M-mode resulted in being a blocked atrial trigeminy with a mean fetal heart rate (FHR) of 100 bpm (Figures 1 and 2). 

A female neonate of 2770 gr and Apgar score 9/10 at 1/5° minutes, respectively, was born by cesarean section at 38 weeks because of the placenta previa. 

**Figure 3:**
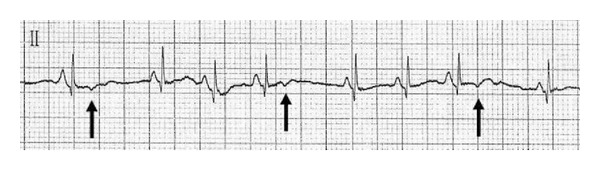
Blocked supraventricular atrial contractions (arrows).

An ECG performed at birth revealed blocked supraventricular extrasystoles (Figure 3). Instead, successively at cardiomonitoring, no extrasystoles were registered.

An ECG the day after did not register any extrasystole. 

On day 3, an ECG showed no atrial extrasystoles and a normal QTc interval at upper limits. An echocardiography performed the same day showed a small patent FO with moderate left-to-right shunt and a trivial tricuspid regurgitation with an indirect estimate of pulmonary artery pressure of 35 mmHg. 

**Figure 4:**
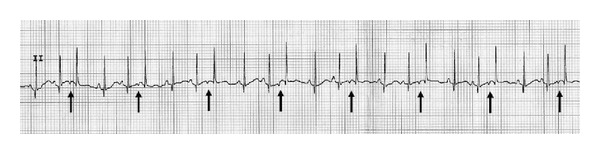
Supraventricular trigeminy (arrows show ectopic atrial beats).

**Figure 5:**
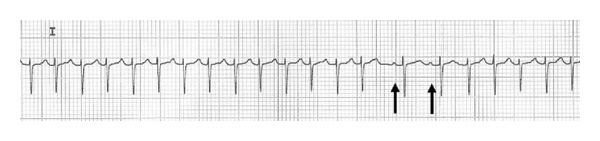
Supraventricular paroxystic tachycardia interrupted by some sinusal beats (arrows).

To our surprise, on day 11, ECG revealed supraventricular trigeminy and episodes of paroxystic supraventricular tachycardia (Figure 4). A treatment with Lanoxin syrup 0.25 mL twice a day was started. A control on day 17 showed a paroxystic supraventricular tachycardia interrupted by some sinusal beats (Figure 5). Digoxinemia level was at 1.4 ng/mL, and therapy with Sotalol hydrochloride 2 mg/kg twice a day was started. 

On day 18, an episode of PSVT that needed a “diving reflex” maneuver was registered. The same day, a cardiomonitoring showed extrasystoles and episodes of bradycardia with a heart rate of 80 bpm. 

A 24 hr ECG/Holter monitoring on day 20 recorded a sinusal rhythm with a mean heart rate at lower limits for age, some blocked supraventricular extrasystoles, and 3 isolated and monomorphic ventricular extrasystoles. During sleep, some episodes of 2nd-degree AV block Mobitz type I and Mobitz type II were recorded. Interventricular conduction was regular. There were no significant alterations of the ventricular repolarization. 

After consulting 24 hr ECG monitoring, we decided to gradually suspend Lanoxin. Currently, the little girl is administered Sotalol hydrochloride 2 mg/kg twice a day and she is doing well. 

## 3. Discussion 

Although most cases of fetal bradycardia verified during obstetrical ultrasound examination are due to vagal stimulation because of fetal compression by the transducer, attention should be paid in case of sustained bradycardias, trying to evaluate simultaneously atrial and ventricular contraction for evidence of nonconducted ectopic atrial beats causing low ventricular contraction rate. Furthermore, these benign arrhythmias can present with bradycardia during the FHR monitoring leading erroneously to emergent preterm delivery. Consequently, in cases when an atypical bradycardia is registered at FHR monitoring, a fetal echocardiography should be done to determine if it is the case of blocked atrial bigeminy or trigeminy, because these arrhythmias do not represent an obstetrical emergency. 

However, the literature shows that although once considered an entirely benign arrhythmia, fetal ectopy is now thought to be a manifestation of a number of diseases. Ectopy should not be dismissed as benign without fetal assessment, especially if risk factors such as a family history of sudden death, prior fetal loss, or maternal pregnancy complications exist [4]. Whether ectopy represents spontaneous automaticity of the atrium or reentry is unclear. When coupling of the ectopic beat to the prior QRS is fixed, as opposed to variable, it is likely to be related to a reentrant atrioventricular pathway; in this setting, the risk of supraventricular tachycardia is about 0.5% for simple ectopy (isolated, bigeminy, or trigeminy) [3, 6] and up to 6% for complex ectopy (atrial couplets or triplets). This increased risk of supraventricular tachycardia extends into the neonatal period, although most ectopy resolves by 1 month of age. 

In our case, the fetus presented with bradycardia due to atrial trigeminy and three subsequent postnatal ECGs until day 3 showed no evidence of atrial extrasystoles, confirming the well-known frequent regression of this kind of benign arrhythmia. But on day 11 recurrence of supraventricular trigeminy and development of episodes of paroxystic supraventricular tachycardia were observed. 

Although our case represents a single evidence, it denotes the risk of development of more serious arrhythmia in postnatal life in cases of fetal blocked atrial ectopic beats. This seems to occur when the underlying mechanism is an accessory AV conduction pathway that appears occult even at basal ECG. 

On the basis of this observation, we recommend that fetuses with complex atrial ectopic beats should be closely monitored before and after birth for evidence of new arrhythmias. Since time period for monitoring is unknown, parents should be advised to verify baby's heart beat during feeding quietness, or sleep and refer both low and high frequencies.
